# Fearfulness associates with problematic behaviors and poor socialization in cats

**DOI:** 10.1016/j.isci.2022.105265

**Published:** 2022-10-03

**Authors:** Salla Mikkola, Milla Salonen, Emma Hakanen, Hannes Lohi

**Affiliations:** 1 Department of Veterinary Biosciences, University of Helsinki, Helsinki, Finland; 2 Department of Medical and Clinical Genetics, University of Helsinki, Helsinki, Finland; 3 Folkhälsan Research Center, Helsinki, Finland

**Keywords:** biological sciences, ethology, zoology

## Abstract

Problematic behavior is a remarkable welfare issue in cats (*Felis catus*), as it is one of the most common reasons for relinquishment. The probability of developing problematic behaviors is likely influenced by several variables, but these remain little studied. In this study, we examined the associations of fearfulness, aggression toward humans, and excessive grooming with nearly thirty variables in a survey dataset of over 3,200 cats. To identify the most important variables influencing these behaviors, we used generalized linear models. All behaviors were associated with each other suggesting comorbidity between problematic behaviors. Breed and several environmental variables were also associated with behaviors. Poor socialization with humans and a history of being a rescue cat were associated with higher fearfulness, indicating that the proper socialization of kittens is beneficial for avoiding fear-related problematic behaviors. Overall, our study highlights the complexity of three problematic behaviors in cats.

## Highlights

• Fearfulness strongly correlated with aggression and excessive grooming
• Irregular visits to a veterinarian were associated with aggression
• Highly human sociable cats had more excessive grooming than less sociable cats
• Poor socialization was associated with fearfulness

## Graphical abstract

**Figure:**
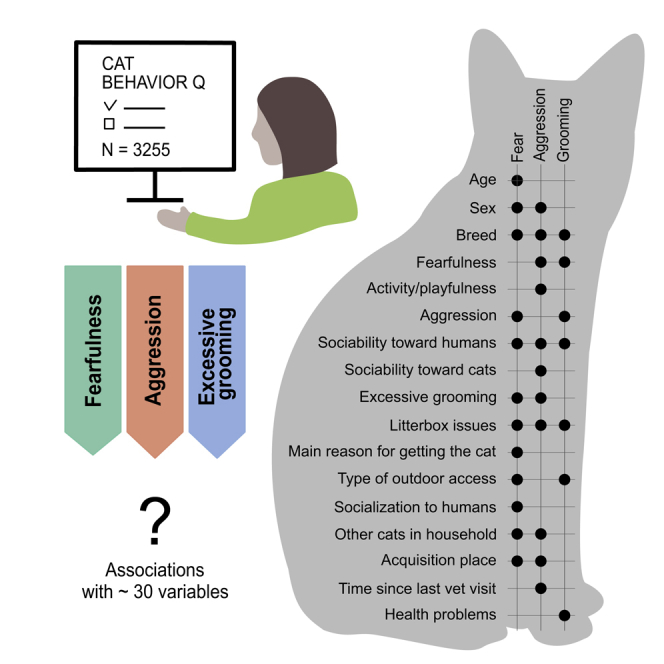

## Introduction

Unwanted or problematic behavior is a remarkable welfare issue in pet cats *(Felis catus)*. Problematic behavior is a common reason for cat relinquishment to an animal shelter (Salman et al., 2010; Stella and Croney, 2016) and may indicate that the cat is experiencing distress or anxiety, which has several negative impacts on welfare (Amat et al., 2009). Fearfulness and aggressiveness are common problematic behaviors (Tamimi et al., 2015; Wassink-van der Schot et al., 2016). Aggressive behavior can include scratching and biting along with hissing and growling. Fearfulness may cause the avoidance of various situations and fear may result in a decreased willingness to approach or interact with people. In addition, the inability to avoid fearful situations may lead to chronic distress (Levine, 2008). Repetitive behaviors, such as excessive grooming, are less common but may be harmful to cats: these can include frantic licking and self-mutilation, for example, pulling out hair, which leads to bald patches (Overall and Dunham, 2002; Bennett and Khan, 2021).

Pet cats may have problematic behavior for several reasons, and previous literature has reported several associations between different factors and problematic behaviors. Examples include age (Naderi et al., 2011; Wassink-van der Schot et al., 2016; Duffy et al., 2017), neutering status (Amat et al., 2009; Arahori et al., 2017), insufficient playing with the cat (Strickler and Shull, 2013), and the opportunity of going outdoors (Amat et al., 2009; Naderi et al., 2011; Tamimi et al., 2015; Ha and Ha, 2017). Furthermore, inadequately socialized kittens have a higher probability of being fearful than normally socialized kittens (McCune, 1995; Casey and Bradshaw, 2008). Company of other cats in the same household has been associated both with lower fearfulness and aggression toward people (Amat et al., 2009; Ahola et al., 2017; Amat and Manteca, 2019; Yamada et al., 2020; Menor-Campos et al., 2021). In addition, genetics may affect the probability of developing these behavioral problems. Previous literature has found occasionally contradictory breed differences between these behaviors (Naderi et al., 2011; Hart and Hart, 2013; Tamimi et al., 2015; Wassink-van der Schot et al., 2016; Ahola et al., 2017; Salonen et al., 2019; Mikkola et al., 2021). Certain personality types are also suggested to be more prone to stress (Foster and Ijichi, 2017) and, thus, to more readily develop problematic behaviors than others. The co-occurrence of multiple problematic behaviors is likely, as e.g. fear-related aggression is quite common in cats (Amat and Manteca, 2019). In addition, health problems, for example, skin diseases and parasites are common causes of excessive grooming (Beale, 2012; Tilley and Smith, 2016).

We collected a convenience sample of over 3,200 Finnish cats using a validated feline behavior and personality survey (Mikkola et al., 2021). This study focused on identifying the risk factors of three problematic behaviors: fearfulness, aggression toward humans, and excessive grooming. Owing to the sampling method used in this study, the problematic behaviors are not clinical diagnoses made by veterinarians, but continuous traits based on the owner’s responses. We studied the associations of these problematic behaviors with 21 environmental and demographic factors, and seven personality and behavioral traits using generalized linear models.

## Results

### Study subjects

The dataset included 3,255 cats, and nearly half of which (49%) were females. Only 11% of the cats were neither sterilized, nor neutered, or were being administered medical contraception. Cat age varied between 3 months and 23 years, with a mean age of 5.9 years. The number of cats within the 26 breed groups ranged from 34 Turkish Vans to 788 Landrace Cat Shorthairs (see Table S1).

### Factors associated with fearfulness

The final generalized linear model for fearfulness included the explanatory variables age, sex, breed, main reason for getting the cat, type of outdoor access, socialization to humans, other cats in household, acquisition place, excessive grooming, litterbox issues, sociability toward humans, and aggression toward humans (Table 1).

**Table 1:** Associations between the explanatory variables and three traits, i.e. fearfulness, aggression toward humans, and excessive grooming, in the generalized linear models

Variable	*DF*	Fearfulness		Aggression toward humans		Excessive grooming	
		*F*	p	*F*	p	*F*	p
Age	1	43.757	**< 0.0001**	1.774	0.2811	3.052	0.2770
Age^2^	1	41.900	**< 0.0001**	2.298	0.2109	0.012	0.9707
Sex	1	9.490	**0.0118**	6.923	**0.0237**	4.208	0.2099
Breed	25	4.256	**< 0.0001**	6.928	**< 0.0001**	2.661	**0.0004**
Fearfulness	1			67.869	**< 0.0001**	29.010	**< 0.0001**
Activity/playfulness	1			38.144	**< 0.0001**		
Aggression toward humans	1	37.097	**< 0.0001**			76.510	**< 0.0001**
Aggression toward humans^2^	1	0.951	0.5079			9.229	**0.0280**
Sociability toward humans	1	180.524	**< 0.0001**	36.103	**< 0.0001**	47.086	**< 0.0001**
Sociability toward cats	1			114.821	**< 0.0001**		
Excessive grooming	1	21.751	**< 0.0001**	128.091	**< 0.0001**		
Excessive grooming^2^	1			61.263	**< 0.0001**		
Litterbox issues	1	46.009	**< 0.0001**	32.648	**< 0.0001**	76.252	**< 0.0001**
Litterbox issues^2^	1	7.953	**0.0239**	11.402	**0.0033**	32.095	**< 0.0001**
Main reason for getting the cat	2	29.626	**< 0.0001**				
Type of outdoor access	4	13.962	**< 0.0001**			6.572	**0.0006**
Socialization to humans	3	10.593	**< 0.0001**				
Other cats in household	3	8.435	**0.0002**	4.541	**0.0115**		
Acquisition place	3	9.787	**< 0.0001**	6.719	**0.0009**		
Time since last vet visit	3			7.261	**0.0005**		
Health problems	2					30.609	**< 0.0001**

Age, aggression toward humans, excessive grooming, and litterbox issues did not meet the linearity assumption in all models, so they were included also as quadratic variables where needed. All p-values are controlled for the false discovery rate. Significant (p < 0.05) associations are emboldened. Symbols <, >, and = symbolize the direction of the effect. *N* = 3,255. See also Table S3.

**Figure 1:**
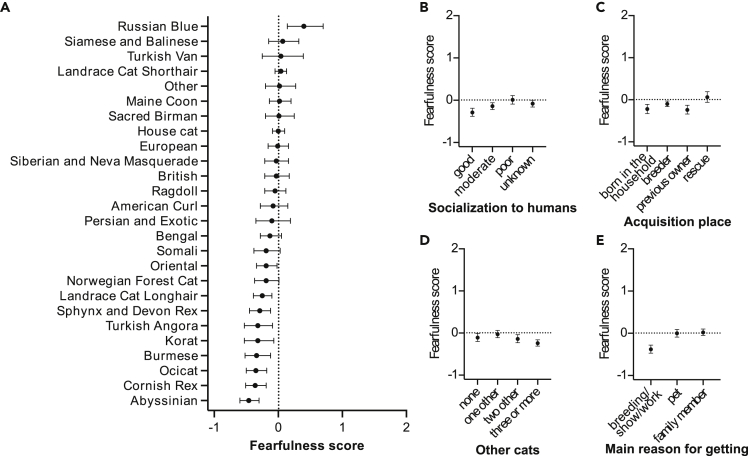
Demographical and environmental factors associated with fearfulness (A–E) Associations of the breeds/breed groups (A), socialization to humans (B), acquisition place (C), other cats in household (D), and main reason for getting the cat (E) with fearfulness in the generalized linear model. Error bars indicate 95% confidence limits. *N* = 3,255. See also Figure S1.

Fearfulness correlated with age, but the effect was non-linear: cats around nine years of age had the highest mean score, and cats younger or older than this had lower scores (see Figure S1A, Table 1). Female cats averaged higher fearfulness score than males (see Figure S1B, Table 2), and breeds differed in their mean scores (Figure 1A, Table 1). The largest difference existed between Russian Blues and Abyssinians, with Russian Blues scoring the highest in fearfulness (Figure 1A). The rest of the pairwise breed differences are presented in Table S2.

**Table 2:** Contrasts between different groups of categorical variables in the generalized linear model analysis of fearfulness

Variable	Contrast	*Z*	p
Sex	female > male	3.095	**0.0118**
Main reason for getting the cat	family member > pet	0.710	0.6644
	family member > breeding/show/work	7.800	**< 0.0001**
	pet > breeding/show/work	6.691	**< 0.0001**
Type of outdoor access	balcony = in a cage or freely supervised	2.066	0.1006
	balcony = freely unsupervised	0.166	0.9286
	balcony > on a leash	6.299	**< 0.0001**
	balcony = none	0.507	0.7598
	in a cage or freely supervised = freely unsupervised	−1.175	0.4009
	in a cage or freely supervised > on a leash	4.569	**< 0.0001**
	in a cage or freely supervised = none	−1.453	0.2809
	freely unsupervised > on a leash	3.857	**0.0011**
	freely unsupervised = none	0.189	0.9250
	on a leash < none	−5.472	**< 0.0001**
Acquisition place	born in the household = breeder	−2.233	0.0764
	born in the household = previous owner	0.250	0.9047
	born in the household < rescue	−3.756	**0.0014**
	breeder > previous owner	2.870	**0.0211**
	breeder < rescue	−3.168	**0.0098**
	previous owner < rescue	−4.935	**< 0.0001**
Socialization to humans	moderate > good	2.974	**0.0158**
	moderate < poor	−3.236	**0.0082**
	moderate = unknown	−1.514	0.2607
	good < poor	−5.489	**< 0.0001**
	good < unknown	−4.267	**0.0002**
	poor = unknown	2.001	0.1123
Other cats in household	none = one other	−1.974	0.1180
	none > three or more	2.702	**0.0282**
	none = two other	0.611	0.7194
	one other > three or more	4.971	**< 0.0001**
	one other > two other	2.585	**0.0373**
	three or more = two other	−2.167	0.0856

All p-values are controlled for false discovery rate. Significant (p < 0.05) associations are emboldened. Symbols <, >, and = symbolize the direction of the effect. *N* = 3,255. See also Table S2.

Many environmental variables were also included as explanatory variables in fearfulness. Early life socialization to humans was associated with fearfulness, with poorly socialized cats averaging higher fearfulness scores than cats socialized well or moderately (Figure 1B and Table 2). Well-socialized cats, on average, also scored lower than cats socialized moderately or cats with unknown socialization to humans. Acquisition place was associated with fearfulness, as rescue cats had a higher mean fearfulness score than cats living in their birth home or cats obtained from previous owners or breeders (Figure 1C and Table 2). In addition, cats obtained from previous owners had a lower mean fearfulness score than cats obtained from breeders. Furthermore, cats living with one other cat had a higher mean fearfulness score than cats living with two or more cats, and cats living alone had a higher mean fearfulness score than cats living with three or more cats (Figure 1D and Table 2). Owner’s motivation for getting the cat was associated with fearfulness; cats obtained as pets or family members had a higher mean fearfulness score than cats obtained for breeding, shows, or work (Figure 1E and Table 2). Finally, cats with outdoor access on a leash had a lower mean fearfulness score than cats with other types of outdoor access (see Figure S1C, Table 2).

**Figure 2:**
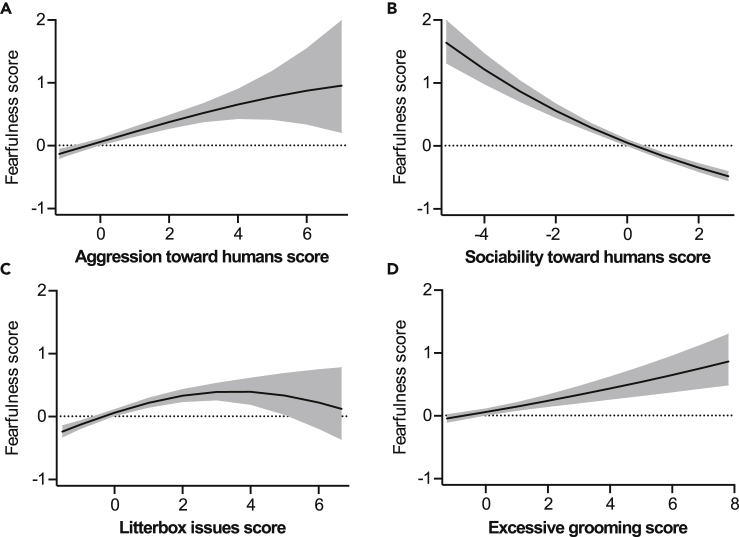
Personality and behavior factors associated with fearfulness (A–D) Associations of aggression toward humans (A), sociability toward humans (B), litterbox issues (C), and excessive grooming (D) with fearfulness in the generalized linear model. Gray area indicates 95% confidence limits. *N* = 3,255. See also Figure S1.

Fearfulness correlated negatively with sociability toward humans (Figure 2B) and positively with excessive grooming (Figure 2D). Correlations with aggression toward humans (Figure 2A) and litterbox issues (Figure 2C) were positive at least until the end of the curve.

### Factors associated with aggression toward humans

The final generalized linear model for aggression toward humans included explanatory variables age, sex, breed, acquisition place, time as last veterinarian visit, fearfulness, sociability toward cats, excessive grooming, activity/playfulness, litterbox issues, other cats in the household, and sociability toward humans (Table 1).

**Figure 3:**
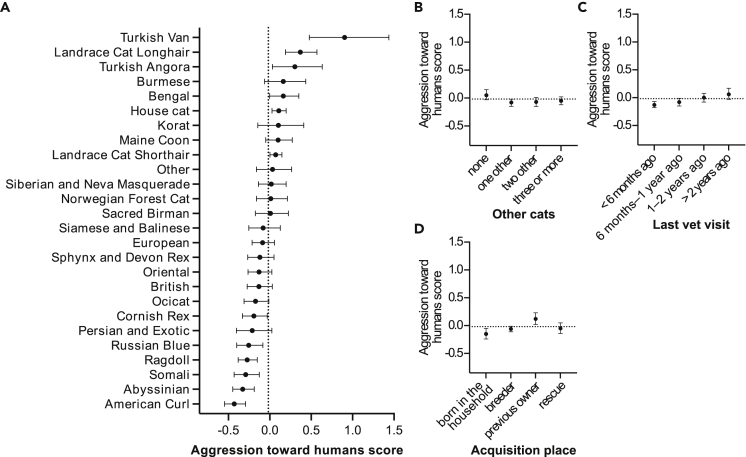
Demographical and environmental factors associated with aggression toward humans (A–D) Associations of the breeds/breed groups (A), other cats in household (B), last vet visit (C), and acquisition place (D) with aggression toward humans in the generalized linear model. Error bars indicate 95% confidence limits. *N* = 3,255. See also Figure S2.

Aggression toward humans did not correlate with age (see Figure S2A, Table 1). Female cats scored higher in aggression toward humans than males (see Figure S2B, Table 3), and breed differences also existed for this trait (Figure 3A, Table 1). The largest difference occurred between the Turkish Van and American Curl breeds, with Turkish Vans scoring the highest (Figure 3A). The remaining pairwise breed differences are found in Table S2.

**Table 3:** Contrasts between different groups of categorical variables in the generalized linear model analysis of aggression toward humans

Variable	Contrast	*Z*	p
Sex	female > male	2.644	**0.0237**
Acquisition place	born in the household = breeder	−1.778	0.1421
	born in the household < previous owner	−4.055	**0.0003**
	born in the household = rescue	−1.651	0.1769
	breeder < previous owner	−3.896	**0.0005**
	breeder = rescue	−0.351	0.7864
	previous owner > rescue	2.934	**0.0111**
Other cats in household	none > one other	3.566	**0.0017**
	none = three or more	2.297	0.0506
	none > two other	2.741	**0.0181**
	one other = three or more	−0.772	0.5512
	one other = two other	−0.279	0.8242
	three or more = two other	0.433	0.7426
Time since last vet visit	6 months–1 year = 1–2 years	−2.010	0.0902
	6 months–1 year = under 6 months	1.365	0.2654
	6 months–1 year < over 2 years	−2.967	**0.0101**
	1–2 years > under 6 months	3.347	**0.0036**
	1–2 years = over 2 years	−1.187	0.3392
	under 6 months < over 2 years	−4.077	**0.0003**

All p-values are controlled for false discovery rate. Significant (p < 0.05) associations are emboldened. Symbols <, >, and = symbolize the direction of the effect. *N* = 3,255. See also Table S2.

Cats living without other cats had a higher mean aggression score toward humans than cats living with other cats (Figure 3B and Table 3). Cats whose previous veterinarian visit was over two years ago scored higher in aggression than cats whose last visit was 6 months to 1 year ago or less than 6 months ago (Figure 3C and Table 3). In addition, cats that were last taken to a veterinarian 1–2 years ago had a higher mean aggression score than cats taken to a veterinarian less than 6 months ago. Finally, cats obtained from previous owners showed higher mean aggression toward humans than cats living in their birth homes or cats obtained from breeders or as rescues (Figure 3D and Table 3).

**Figure 4:**
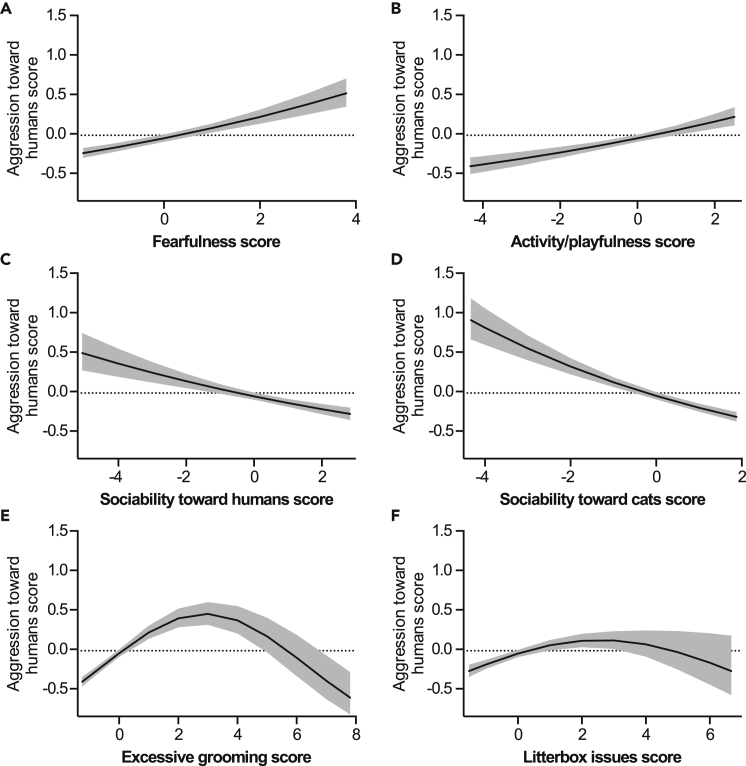
Personality and behavior factors associated with aggression toward humans (A–F) Associations of fearfulness (A), activity/playfulness (B), sociability toward humans (C), sociability toward cats (D), excessive grooming (E), and litterbox issues (F) with aggression toward humans in the generalized linear model. Gray area indicates 95% confidence limits. *N* = 3,255. See also Figure S2.

Aggression toward humans correlated positively with fearfulness and activity/playfulness, and negatively with sociability toward humans and cats (Figures 4A–4D). Correlations with aggression toward humans and excessive grooming and litterbox issues were non-linear (Figures 4E and 4F).

### Factors associated with excessive grooming

The final generalized linear model for excessive grooming included the explanatory variables age, sex, breed, health problems, type of outdoor access, aggression toward humans, litterbox issues, sociability toward humans, and fearfulness (Table 1).

**Figure 5:**
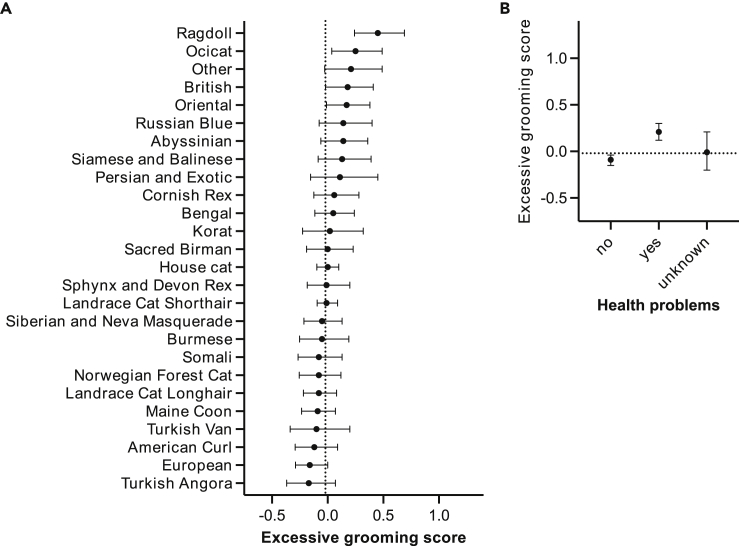
Demographical and environmental factors associated with excessive grooming (A and B) Associations of the breeds/breed groups (A) and owner-reported health problems (B) with excessive grooming in the generalized linear model. Error bars indicate 95% confidence limits. *N* = 3,255. See also Figure S3.

Excessive grooming was not associated with age or sex (see Figures S3A and S3B, Table 1). Breeds also differed in this trait, and the largest difference occurred between Ragdoll and Turkish Angora, with Ragdoll having the highest mean excessive grooming score (Figure 5A). Cats with owner-reported health problems had a higher mean excessive grooming score than cats without reported health problems (Figure 5B and Table 4). Finally, cats with freely unsupervised access to the outdoors had a lower mean excessive grooming score than cats without any outdoor access, cats with only access to a balcony, or cat with outdoor access on a leash (see Figure S3C, Table 4). Furthermore, cats with outdoor access in a cage or freely supervised had a lower mean excessive grooming score than cats with access only to a balcony or only on a leash.

**Table 4:** Contrasts between different groups of categorical variables in the generalized linear model analysis of excessive grooming

Variable	Contrast	*Z*	p
Sex	female = male	2.050	0.2099
Health problems	no = unknown	−0.863	0.6657
	no < yes	−7.744	**< 0.0001**
	unknown = yes	−1.874	0.2429
Outdoor access	balcony > in a cage or freely supervised	2.859	**0.0450**
	balcony > freely unsupervised	4.297	**0.0004**
	balcony = on a leash	0.401	0.8529
	balcony = none	1.187	0.5021
	in a cage or freely supervised = freely unsupervised	2.534	0.0965
	in a cage or freely supervised < on a leash	−2.890	**0.0421**
	in a cage or freely supervised = none	−1.484	0.3895
	freely unsupervised < on a leash	−4.264	**0.0005**
	freely unsupervised < none	−3.376	**0.0107**
	on a leash = none	0.945	0.6207

All p-values are controlled for false discovery rate. Significant (p < 0.05) associations are emboldened. *N* = 3,255. See also Table S2.

**Figure 6:**
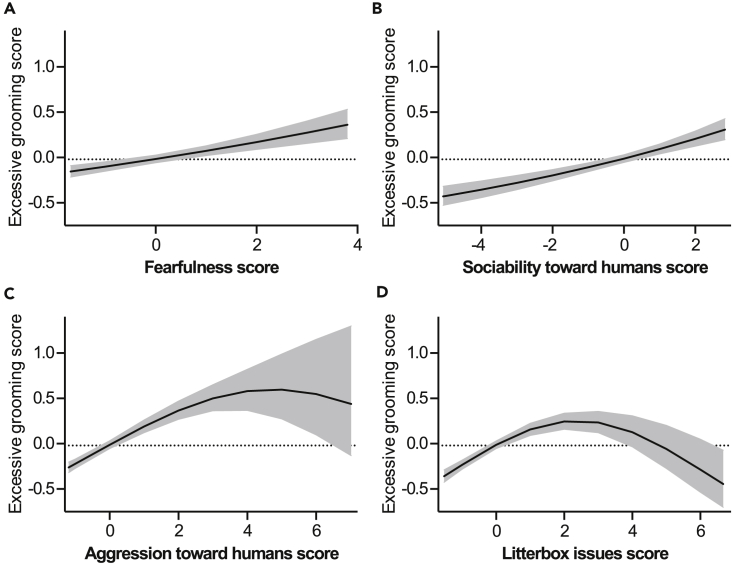
Personality and behavior factors associated with excessive grooming (A–D) Associations of fearfulness (A), sociability toward humans (B), aggression toward humans (C), and litterbox issues (D) with excessive grooming in the generalized linear model. Gray area indicates 95% confidence limits. *N* = 3,255. See also Figure S3.

Excessive grooming correlated positively with fearfulness and sociability toward humans (Figures 6A and 6B). Correlations with excessive grooming, aggression toward humans and litterbox issues were non-linear (Figures 6C and 6D).

## Discussion

Understanding the factors that predispose cats to behavioral problems is essential for developing better management approaches and improving feline welfare. Utilizing a validated survey sample of over 3,200 cats, we identified various demographic, environmental, personality, and behavioral factors that may contribute to fearfulness, aggression toward humans, and excessive grooming. Each of the three behavioral problems was statistically associated with several contributing factors, including e.g., socialization to humans, presence of conspecifics, health problems, and litterbox issues.

Nearly every personality and behavioral trait correlated with fearfulness, aggression, or excessive grooming. Fearful cats were more aggressive and expressed more excessive grooming than less fearful cats, paralleling previous studies (Feaver et al., 1986; Ahola et al., 2017). Aggression caused by fear is a common phenomenon in cats (Amat and Manteca, 2019), which could explain the first connection. Excessive grooming can be induced by stress or trauma (Overall and Dunham, 2002; Luescher, 2003) and fearful cats may be more vulnerable to it, which could explain the second connection.

We discovered that cats sociable toward humans were less fearful and aggressive but more vulnerable to excessive grooming. Fearfulness may inhibit a willingness to approach people, especially strangers, which could explain the connection. Correlations between fearfulness, sociability, and aggression toward humans have been found previously (Feaver et al., 1986). The association between sociability toward humans and excessive grooming has not been found before. It may suggest that strongly human-oriented cats are more dependent on human company. Excessive grooming is also a sign of separation-related problems (Schwartz, 2002; de Souza Machado et al., 2020), possibly indicating that separation-related issues may be more common in cats that are more sociable toward humans. However, this requires further investigation.

Litterbox issues correlated positively with fearfulness, and non-linear associations existed with aggression and excessive grooming. Litterbox issues can be triggered by anxiety (Herron, 2010), which may be more common in fearful individuals. Interestingly, moderate scores in litterbox issues were associated with the highest scores in aggression and excessive grooming. Aggression toward humans and excessive grooming formed a similar pattern, and this has also been found previously (Ahola et al., 2017). On the other hand, non-linear connections between aggression toward humans, excessive grooming, and litterbox issues maybe anomalies owing to the low number of cats exhibiting high scores in those traits.

Activity/playfulness only correlated with aggression toward humans, with active and playful cats having higher mean scores in aggression. This connection may be a consequence of misdirected predatory behavior, which is a very common cause of aggression toward human family members in cats (Amat and Manteca, 2019).

In addition to personality and behavioral traits, several environmental factors were associated with fearfulness, aggression, and excessive grooming. However, their individual effect on the problematic behavioral trait was small in many cases, and causality remained unclear. For example, cats obtained for breeding, shows, or work were less fearful than cats obtained as family members or pets. Individual differences in motor activity and behavior can be seen in kittens even before weaning (Raihani et al., 2014), so a likely explanation for this finding is that breeders may have selected the least fearful kittens for breeding purposes and sold the rest of the litter as pets or family members to new owners.

Socialization of humans in kittenhood was negatively associated with fearfulness. Poorly socialized cats were more fearful than well or moderately socialized cats. Thus, it seems beneficial for cats to meet unfamiliar adults and children at least weekly during 0–12 weeks of age. In addition, cats with unknown socialization levels to humans were more fearful than well-socialized cats. This unknown group included a considerable proportion of rescue cats, and their socialization to humans as kittens was likely poor. Only a few previous studies have investigated the effect of socialization on cat’ behavior. Although these studies were conducted with shelter- and laboratory-reared cats, they similarly suggest that handling kittens during the early socialization period is beneficial (McCune, 1995; Lowe and Bradshaw, 2002; Casey and Bradshaw, 2008). It is also hypothesized that well-socialized cats endure negative experiences better and trust their new guardians sooner than poorly socialized cats (Turner, 2021). Furthermore, inadequate socialization is named as the main environmental cause of fear-related aggression (Amat and Manteca, 2019).

Rescue cats were more fearful than cats living in their birth homes or cats obtained from breeders or previous owners. Interestingly, cats acquired from breeders were more fearful than cats obtained from previous owners. However, pre-owned cats expressed the most aggression toward humans. As aggressive behavior is one of the most common reasons for relinquishing a cat (Salman et al., 2010), aggressiveness was probably the reason for the previous owner seeking a new home for their cat.

Cats that had last seen a veterinarian over two years ago or never were more aggressive than cats that had visited a veterinarian less than one year ago. A minimum annual wellness examination is recommended for cats by veterinarians and veterinary organizations (Hoyumpa Vogt et al., 2017). However, owners may avoid visits to a veterinarian if they know their cat will behave aggressively during a health examination or if they consider health examinations stressful for their cat (Karn-Buehler and Kuhne, 2021). However, it is also possible that cats that rarely visit the veterinarian may have undiagnosed health problems that cause pain, leading to aggressive behavior (Amat and Manteca, 2019; Mills et al., 2020). Indeed, poor pain management and not seeking veterinary care are known welfare issues (Rioja-Lang et al., 2019). In addition, cats with owner-reported health problems expressed more excessive grooming than cats without them. Excessive grooming is not usually a compulsive behavior, and it can be a sign of various skin conditions or a food allergy (Tilley and Smith, 2016).

The number of other cats in a household was associated with fearfulness and aggression toward humans. Cats living alone or with one cat were more fearful than cats living with multiple cats. Similarly, Yamada et al. (2020) found that the company of other cats is beneficial for fearful cats. Furthermore, we discovered that cats without conspecifics were slightly more aggressive than cats living with one or two other cats, but there was no difference compared with cats living with three or more conspecifics. Most previous studies have found less aggression toward human family members or humans in general in multi-cat households (Amat et al., 2009; Ahola et al., 2017; Amat and Manteca, 2019; Yamada et al., 2020; Menor-Campos et al., 2021). The presence of other cats may enable interspecific play and communication, which is likely important for the majority of cats. On the other hand, this association can indicate that owners of fearful or aggressive cats may be unwilling to get more cats, as they predict that the situation may worsen. For example, getting more cats increases the probability of aggressive behavior between the cats (Elzerman et al., 2020) and, overall, can either increase or decrease their welfare (Finka and Foreman-Worsley, 2022).

Breed influenced fearfulness, aggression toward humans, and excessive grooming. The Russian blue was the most fearful breed and the Abyssinian the least fearful. The Turkish Van expressed the most aggression toward humans, and the American Curl expressed the least aggression. The Ragdoll scored the highest in excessive grooming, and the Turkish Angora scored the lowest. Most breeds did not differ in the pairwise comparisons, and individual differences within a breed were large.

The behavioral profiles of the breeds are quite similar to previous studies (Amat et al., 2009; Takeuchi and Mori, 2009; Hart and Hart, 2013; Tamimi et al., 2015; Wilhelmy et al., 2016; Duffy et al., 2017; Hart et al., 2018; Salonen et al., 2019). For example, the Turkish Van has been ranked as the most aggressive breed in previous studies of local cat population (Vapalahti et al., 2016; Salonen et al., 2019). Breed order differs only slightly from a previous study that partly used the same data but did not include other factors in the models (Mikkola et al., 2021). Interestingly, short- and long-haired Landrace Cats differ from each other, even though both can be born in the same litter. Short-haired Landrace Cats were more fearful than long-haired cats, while long-haired individuals expressed more aggression toward humans. Higher aggression in long-haired cats may be a pure effect of their longer fur, which tangles more easily than short fur, as grooming out the tangles can be painful. Another possibility is that the gene regions affecting fur length may be linked to genes affecting personality.

In conclusion, this study indicates that feline fearfulness, aggression toward humans, and excessive grooming are affected by personality and behavioral traits and by numerous environmental and demographic factors. In general, the individual effects of the environmental variables were smaller than the effects of personality, behavior, or breed. Fearfulness in particular seemed an important personality trait that affects other personality dimensions. It was associated with other problematic behavioral traits in the study: aggression toward humans, excessive grooming, and litterbox issues. As fearfulness was associated with socialization in humans, we may expect that proper socialization is also beneficial for avoiding other problematic behaviors. In addition, as fearfulness is heritable (Salonen et al., 2019), breeders may, in theory, decrease the average fearfulness by preferring non-fearful parents in breeding. However, signs of fear in cats are much harder to identify than aggression, at least for the owners (Karn-Buehler and Kuhne, 2021), and no easy and effective tools are available for breeders to evaluate the fearfulness or other personality traits of their breeding individuals.

Furthermore, our results suggest that owning multiple cats may decrease the probability of aggression toward humans. In addition, our results may indicate that cats expressing high sociability toward humans are more vulnerable to excessive grooming than less social individuals. However, longitudinal studies are needed to investigate our findings further.

### Limitations of the study

Our study has limitations. Owing to the cross-sectional nature of this study, the achieved results are correlations and associations rather than causations. We could not identify the reasons behind the behaviors. For example, we do not know if cats who received high scores in excessive grooming had psychogenic alopecia or some other health problems that cause itching or discomfort. In addition, we needed to transform originally numerical information into class variables and unite certain categories together to reach reasonable group sizes. Some breeds, such as the Turkish Van, had low sample sizes. Furthermore, all traits did not meet the linearity assumption. We also needed to drop certain variables owing to high multicollinearity during the analyses. In addition, certain interesting and probably important variables, such as more detailed information concerning the socialization period, had a high proportion of missingness, and we were unable to use the information more precisely. Furthermore, as the survey was answered by current owners rather than the breeders, information regarding the time prior to weaning and arrival at the current owner may be inaccurate.

## STAR★Methods

### Key resources table

**Table:** 

REAGENT or RESOURCE	SOURCE	IDENTIFIER
**Deposited data**		

Data used in the study	Figshare	https://doi.org/10.6084/m9.figshare.21118915

**Software and algorithms**		

Code used in the study	Mendeley Data	https://doi.org/10.17632/472vnjvb2m.1
R 4.1.1	R Core Team	https://cran.r-project.org/bin/windows/base/

### Resource availability

#### Lead contact

Further information and requests should be directed to and will be fulfilled by the lead contact, Hannes Lohi (hannes.lohi@helsinki.fi).

#### Materials availability

This study did not generate new unique reagents.

### Experimental model and subject details

Participants i.e., the cat owners, had to be at least 18 years old to be able to fill the survey, but the exact age of the participants was not collected. Participants’ sex and gender identity was not collected. The data set used in this study included 3,255 cats, but the participants were able to fill the survey for every cat they had. The study was conducted according to the guidelines of the Declaration of Helsinki and approved by the University of Helsinki Viikki Campus Research Ethics Committee (11.02.2019). Informed consent was obtained from all respondents.

### Method details

#### Questionnaire, data, and scores

The feline behaviour and personality survey used during data collection was published in Mikkola et al. (2021). The survey included basic demographic information concerning the cats, along with three separate sections: behaviour and personality, background, and health. The data were collected between March 2019 and September 2020 and were partially published in Mikkola et al. (2021). The published data set included basic demographic information of the cats and the behaviour and personality section of the survey. In contrast, the main data used in this article are from the background and health sections of the survey.

The seven personality and behaviour traits used in this study were previously extracted and named fearfulness, activity/playfulness, aggression toward humans, sociability toward humans, sociability toward cats, litterbox issues and excessive grooming (Mikkola et al., 2021). Factor scores were extracted with a correlation-preserving method (tenBerge). Therefore, each cat received a score for each trait based on its owner’s response. For this study, we utilized the personality and behaviour scores along with the demographic factors of age, sex, and breed group from the published data set (Figshare: https://doi.org/10.6084/m9.figshare.14899077.v2).

The fearfulness factor included 19 items, for example, “growls or hisses when an unfamiliar person tries to touch or pet him/her” and “is easily scared even by small, unexpected things and sounds”. Aggression toward humans included 17 items, for example, “attempts to scratch or bite when being brushed” and “attempts to scratch or bite when given medicine by a familiar person”. Excessive grooming included three items, “shows excessive and intensive grooming (inhibits other behaviours) throughout the day”, “exhibits self-mutilation, e.g., pulls hairs off with teeth, vigorously nibbles or bites his/her body parts”, and “exhibits sudden frantic licking or chewing his/her body”. The activity/playfulness factor included items related to playing, running, and jumping, sociability toward cats included seeking and enjoying the company of other cats, the sociability toward humans included purring, seeking attention from humans and separation problems, and litterbox issues included items related to inappropriate elimination and substrate preference. All item loadings can be found in Mikkola et al. (2021).

#### Construction of explanatory variables

The data set included over 30 questions related to the cat’s background and current environment. We formed 17 new variables from these questions: hormonal status, number of siblings, socialization to humans, socialization to animals, place where the cat acquired from (acquisition place here after), main reason for getting the cat, food type, feeding style, number of large scratching trees, number of small scratching trees, playtime frequency, type of outdoor access, average number of days the cat is left alone during the week, hobbies, number of other cats in household (other cats in household here after), and owner’s previous cat experience/ownership (see Table S3). Some initially numeric variables, such as the number of siblings, had to be transformed into categories due to missing values. Similarly, we had to combine certain groups. For example, the “rescue” group included cats adopted from an Animal Welfare Association/shelter and cats taken directly from e.g. barns or the street.

In addition, we combined certain questions into new variables. We combined the questions “how often the cat met unfamiliar adults at the age of 0–12 weeks” and “how often the cat met unfamiliar children at the age of 0–12 weeks” into the ‘socialization to humans’ variable. The new categories were “poor” (the cat met both unfamiliar adults and children on a couple of occasions or not at all); “moderate” (the cat met unfamiliar adults weekly or daily, but children on only a couple of occasions or not at all, or vice versa, or one question was left blank while the response to the other question was weekly or daily); “good” (the cat met both unfamiliar adults and children weekly or daily); and “unknown” (both questions were unanswered or the response to one question was answered not at all while the other question was left blank). In socialization to animals, we combined the questions “how often the cat met unfamiliar cats at the age of 0–12 weeks”, “how often the cat met unfamiliar dogs at the age of 0–12 weeks”, and “how often the cat met other animals at the age of 0–12 weeks” and we then formed the categories “yes” (the cat met unfamiliar dogs, cats, or other animals on at least a couple of occasions), “no” (the cat had not met unfamiliar dogs, cats, or other animals), and “unknown” (all questions were unanswered or the response to some questions was not at all while other questions were left blank).

The variable food type combined information from a multiple-choice question where we asked respondents what types of food their cat eats. Response options were raw meat, raw bones and cartilage, cooked meat, wet food, dry food, prey animals, and something else. If, for example, raw meat was chosen, the following options were used to ascertain how often the cat eats raw meat: as a main food, daily, weekly, and less often. We marked all unselected food types as “never”. We formed groups “dry food only” (dry food was given as the main food or daily, and other food types were given weekly or less often), “dry as the main food” (dry food was given as the main food, and at least one other food type was given as the main food or daily), “dry food daily “(dry food was given daily, and at least one other food type was given as the main food or daily), “no dry food daily” (dry food was given weekly, less often, or never, and other food types were given as the main food, either daily, weekly, less often, or never).

The variable type of outdoor access was also formed from a multiple choice question. We asked each owner whether their cat is allowed to go outdoors and owners were prompted to select all suitable options from: “freely unsupervised”, “freely supervised”, “on a leash”, “in a fenced backyard or outdoor cage”, “on a balcony”, and “my cat does not have access to the outdoors”. To simplify this information, we formed the following groups: “none” (cat did not have access to the outdoors), “balcony” (cat only had access to a balcony), “on a leash” (cat had access to the outdoors only while on a leash or had access both on a leash and to a balcony), “in a cage or freely supervised” (cat had access to the outdoors in a fenced backyard, outdoor cage, or freely but supervised, with or without access to a balcony or on a leash), and “freely unsupervised” (cat had access to the outdoors freely unsupervised, with or without any other access type).

Finally, we enquired about possible diseases and other health problems the cat may have. The health information was simplified into three groups: “yes”, “no”, or “unknown”. A cat was included in the “yes” group if the disease was severe and may have influenced its daily life and behaviour (see Table S4). If the owner had not filled in the health section of the survey, the cat was included in the “unknown” group. We additionally asked “when was the last time your cat was taken to a veterinarian?” and formed the groups: “less than 6 months ago”, “6–12 months ago”, “1–2 years ago,”, and “over 2 years ago”. Cats that had never visited a veterinarian (*N* = 14) or whose owners had not filled in the health section were placed in the “over 2 years ago” group.

### Quantification and statistical analysis

We used generalized linear models to examine the behavioural, demographic, and environmental factors associated with fearfulness, aggression toward humans, and excessive grooming. The data set used in this study included 3,255 cats. We constructed three separate models, one for each trait. The traits were non-normally distributed, and therefore we chose more suitable distributions for them using the ‘rcompanion’(Mangiafico, 2019) and ‘boot’(Canty and Ripley, 2021) packages. The gamma distribution with the log-link function was the best distribution for all.

We selected important explanatory variables with 5-fold cross-validation. First, we divided the data into five data sets (*N* = 2,603–2,605), which each included ∼80% of the cats, with the ‘caret’ package (Kuhn, 2021). For each data set, we chose the best models with the forward stepwise Akaike Information Criterion (AIC) selection approach using the ‘airGLMs’ package (Niskanen et al., 2021). We obtained five different models for each trait. We selected the variables occurring in every model of the specific trait (see Table S5). For example, feeding style occurred in only two of the models in fearfulness and was thus not part of the final fearfulness model. In addition, we calculated relative influences for all the variables (see Table S5) using the ‘gbm’ package (Greenwell et al., 2020). The number of trees was set to 10,000 and the interaction depth to 1 in all the models. Shrinkage was set to 0.003 and the bag fraction to 0.5 in the fearfulness and aggression toward humans models while the same criteria were set to 0.002 and 0.7 in the excessive grooming model, respectively. We decided to exclude variables if their relative influences were poor. For example, the variables hormonal status and hobby were included in all fearfulness models, but their relative influences were low, and we therefore, excluded them from the final fearfulness model.

We used generalized additive models with the ‘gam’ package (Hastie, 2020) to inspect the linearity assumption of the continuous variables in the final models. In the final model of fearfulness, the variables age, litterbox issues, excessive grooming and aggression toward humans did not meet the linearity assumption, and all apart for excessive grooming were added as both linear and quadratic variables into the final model. Adding excessive grooming as a quadratic variable further decreased the linearity, and it was thus not added into the final model. The variables age, excessive grooming, litterbox issues, and sociability toward cats did not meet the linearity assumption in the aggression toward humans model, so we included them all as linear and quadratic variables in the final model, except sociability toward cats. Again, adding sociability toward cats as a quadratic variable decreased the linearity, and thus we did not add it. In the excessive grooming model, the variables age, aggression toward humans, and litterbox issues did not meet the linearity assumption, so we included all of them as linear and quadratic variables in the final model.

After this, we visually inspected the residuals of the final models using the ‘rcompanion’ (Mangiafico, 2019) and ‘boot’ (Canty and Ripley, 2021) packages and possible outliers with the ‘broom’ (Robinson et al., 2021), ‘dplyr’ (Wickham et al., 2021) and ‘ggplot2’ packages (Wickham, 2016). We found several outliers and compared the results of data sets that included or excluded these outliners. Removing the outliers did not change the results significantly, and we kept them in the data because they were real observations. We calculated the variance inflation factor (VIF) with the ‘car’ package (Fox and Weisberg, 2019) to evaluate multicollinearity and did not detect significant multicollinearity. Then we inspected the general fit with the Durbin-Watson test from the ‘lmtest’ package (Zeileis and Hothorn, 2002), and found it to be good.

We conducted an analysis of variance (ANOVA) with the ‘car’ package (Fox and Weisberg, 2019) to see the overall effect of all the variables. Then we used the ‘emmeans’ package (Lenth, 2021) to calculate the estimated marginal means for categorical and binary variables, and the ‘effects’ package (Fox, 2003; Fox and Weisberg, 2019) to obtain the means and confidence limits of the continuous variables. Finally, we corrected the obtained p-values for the false discovery rate (FDR). The significance cut-off was set at p-value < 0.05. R 4.1.1 (R Core Team, 2021) was used in all analyses.

## Data Availability

• The data have been deposited at Figshare and are publicly available as of the date of publication. Accession numbers are listed in the key resources table. • All original code has been deposited at Mendeley Data and is publicly available as of the date of publication. DOIs are listed in the key resources table. • Any additional information required to reanalyze the data reported in this paper is available from the lead contact upon request.

## Supplemental information

Document S1. Figures S1–S3 and Tables S1 and S3–S5

Table S2. Breed contrasts of fearfulness, aggression toward humans, and excessive grooming, related to Tables 1–4 All p-values are controlled for the false discovery rate. Significant (p < 0.05) associations are emboldened. N = 3,255
