# Supplementary material for: Fearfulness associates with problematic behaviors and poor socialization in cats
Source: iScience. 2022 Oct 3;25(10):105265. doi: 10.1016/j.isci.2022.105265 (PMC9579021; doi:10.1016/j.isci.2022.105265)
Supplement: Document S1. Figures S1–S3 and Tables S1 and S3–S5 [file mmc1.pdf]

iScience, Volume *25*

Supplemental information

Fearfulness associates with problematic behaviors and poor socialization in cats

Salla Mikkola, Milla Salonen, Emma Hakanen, and Hannes Lohi

Table S1. Breeds and breed groups with basic demographic information, related to Table 1.

Breed	Age mean (years)	Female intact %	Female sterile %	Male intact %	Male sterile %	*N*
Abyssinian	5.20	9	37	10	45	82
American Curl	5.41	15	28	19	37	67
Bengal	5.66	5	45	3	47	116
British	4.55	11	48	4	37	90
Burmese	5.32	4	53	6	38	53
Cornish Rex	6.34	12	35	5	48	82
European	5.53	20	42	8	30	133
House cat	6.90	2	46	1	51	558
Korat	7.12	5	41	3	51	39
Landrace Cat Longhair	5.55	2	52	0	46	125
Landrace Cat Shorthair	6.38	2	45	1	53	788
Maine Coon	4.60	16	27	8	49	124
Norwegian Forest Cat	5.89	8	37	4	51	76
Ocicat	5.87	9	41	7	43	87
Oriental	5.59	6	41	2	51	102
Other	3.97	10	38	2	50	60
Persian and Exotic	6.05	5	32	8	54	37
Ragdoll	5.20	12	41	3	45	120
Russian Blue	4.21	9	38	5	48	64
Sacred Birman	5.32	5	42	2	52	66
Siamese and Balinese	5.96	5	42	2	52	62
Siberian and Neva Masquerade	4.48	15	32	8	45	98
Somali	6.05	5	52	3	40	63
Sphynx and Devon Rex	5.25	21	40	7	32	85
Turkish Angora	3.22	20	25	16	39	44
Turkish Van	7.87	9	41	3	47	34
Total	5.87	7	42	4	47	3255

Table S3. Variables, their explanations, and categories for the class variables and median and range for the numerical variables derived and modified from the feline behaviour and personality survey, related to Table 1.

Variable	Type	Explanation	Categories/ median[range]
Age	Numerical	Age in years at the time of the participation.	5.0[0.3 - 22.7]
Aggression toward humans	Numerical	Describes aggressiveness toward humans. Highest loading items: cat attempts to scratch or bite when his/her nails are clipped and cat attempts to scratch or bite when medicine by a familiar person.	-0.4[-1.2 - 7.0]
Fearfulness	Numerical	Describes fearfulness toward humans and situations. Highest loading items: cat escapes or hides from unfamiliar people and is comfortable and cat is relaxed among people in social gatherings (e.g. parties)(negatively).	-0.2[-1.7 - 3.8]
Activity/playfulness	Numerical	Describes playfulness and activity. Highest loading items: cat stalks, chases or pounces on moving objects (e.g., string, balls, soft toys), cat runs around in the house while playing and cat is active, does not spend long time idle.	0.2[-4.3 - 2.5]
Sociability toward cats	Numerical	Describes sociability toward cats. Highest loading items: seeks company of other cats in the household (choose "I don't know" if she/he is the only cat in the household) and seeks physical contact from other cats in the household, e.g., sleeps next to them or grooms them (choose "I don't know" if she/he is the only cat in the household).	0.2[-5.0 - 1.9]
Sociability toward humans	Numerical	Describes sociability toward humans. Highest loading items: often seeks out physical contact from people, nudges or nuzzles and always purrs when petted.	0.1[-5.1 - 2.8]
Litterbox issues	Numerical	Describes litterbox use and litter preferences. Highest loading items: urinates (crouching position) in inappropriate places and defecates in inappropriate places indoors.	-0.3[-1.5 - 6.7]
Excessive grooming	Numerical	Describes excessive grooming. Highest loading items: cat shows excessive and intensive grooming (inhibits other behaviours) throughout the day and exhibits self-mutilation, e.g., pulls hair off with teeth, vigorously nibbles or bites his/her body parts.	-0.3[-1.2 - 7.8]
Sex	Binary	Sex of the cat.	female, male
Hormonal status	Binary	Hormonal status of the cat. The sterile category included both physically and chemically sterilized cats.	intact, sterile
Breed	Categorial	Breed or breed group of the cat.	Abyssinian, American Curl, Bengal, British, Burmese, Cornish Rex, European, House cat, Korat, Landrace Cat Longhair, Landrace Cat Shorthair, Maine Coon, Norwegian Forest Cat, Ocicat,

			Oriental, Other, Persian and Exotic, Ragdoll, Russian Blue, Sacred Birman, Siamese and Balinese, Siberian and Neva Masquerade, Somali, Sphynx and Devon Rex, Turkish Angora, Turkish Van
Acquisition place	Categorial	Acquisition place of the cat. The rescue category included cats adopted from an Animal Welfare Association/shelter as rescues as well as cats obtained directly from barns/streets.	rescue, breeder, previous owner, born in the household
Siblings	Categorial	Number of siblings.	none, 1-2, 3, 4, 5 or more, unknown
Socialization to humans	Categorial	Amount of socialization to humans as a kitten until the age of 12 weeks.	poor, moderate, good, unknown
Socialization to animals	Categorial	Describes did the cat met unfamiliar dogs, cats, or other animals as a kitten until the age of 12 weeks.	yes, no, unknown.
Food types	Categorial	Describes the diet.	dry food only, dry as a main food, dry food daily, no dry food daily
Large scratching trees	Categorial	Number of large scratching trees in the home.	none, one, two or more
Small scratching trees	Categorial	Number of small scratching trees in the home.	none, one, two or more
Feeding style	Binary	Describes the style the cat is fed with. The category "from a bowl" included also the use of automatic feeders and the category "active methods" all active feeding methods e.g., feeding puzzles. If both methods were used the cat was grouped to "active methods" category.	from a bowl, active methods
Main reason for getting the cat	Categorial	Owner’s main reason for getting the cat.	family member, pet, breeding/show/work
Owner's previous cat experience/ownership	Categorial	Number of the cats the owner has had before this cat.	none, 1, 2, 3 or 4, 5 or more
Other cats in household	Categorial	Number of other cats in the household.	none, one other, two other, three or more other
Days the cat is left alone during the week	Categorial	Number of days the cat is left alone during the week in average.	0-2, 3 or 4, 5, 6 or 7
Type of outdoor access	Categorial	Describes the outdoor access type available. For example, if the cat had access outdoors both in a leash and freely supervised it was categorized to "freely supervised".	none, balcony, on a leash, in a cage or freely supervised, freely unsupervised
Playtime frequency	Categorial	Describes how often the owner plays with the cat.	several times during a day, daily, several times in a week, weekly, less than weekly
Hobby	Binary	Describes does the owner hobby with the cat. Cats with cat shows as their only hobby were grouped to the "no" category.	yes, no

Time since last vet visit	Categorial	The last time the cat was taken to a veterinarian.	less than 6 months ago, 6 months - 1 year ago,<br>1 - 2 years ago, over 2 years ago
Health problems	Categorial	Describes did the cat had significant owner-reported health problems at the time of participation.	yes, no, unknown

Table S4. Owner-reported diseases and health problems and their classification, related to Table 4. Classification was based on an evaluation how much the disease affects the behaviour of the cat.

Health problem	Criterion(s) for "yes" group	Criterion(s) for "no" group
Acute inflammatory bowel disease	At the moment	Less than half a year ago or more than half a year ago
Amputation	Amputation of legs, several toes, or whole tail	Amputation of only one toe or tip/part of the tail
Anal gland problem	At the moment	Less than half a year ago or more than half a year ago
Asthma	Daily	Weekly or less than weekly
Atopy	At the moment	Less than half a year ago or more than half a year ago
Bald patches	At the moment	Less than half a year ago or more than half a year ago
Bladder stones	At the moment	Less than half a year ago or more than half a year ago
Blind	Any	-
Blocked tear duct	Causing symptoms at the moment	Causing symptoms less than half a year ago or more than half a year ago
Chronic inflammatory bowel disease	Any	-
Deaf	Deaf from both ears	Deaf from just one ear
Dental calculus	At the moment and a lot	Less than half a year ago or more than half a year ago or at the moment and moderately or a little
Diabetes mellitus	Causing symptoms at the moment	Caused symptoms less than half a year ago or more than half a year ago
Ear infection	At the moment	Less than half a year ago or more than half a year ago
Epilepsy	Daily, weekly, couple times in a month or monthly	Less often than monthly
External parasites	Less than a week ago and at least one parasite selected: *Demodex* mites, earmites, lice, fleas, *Cheyletiella* mites, *Notoedres* mites	Less than a week ago, half a year ago or more than half a year ago and selected ticks only or less than half a year ago or more than half a year ago and selected any other parasite(s)
Eye infection	At the moment	Less than half a year ago or more than half a year ago
Eye/eyes removed/missing	Both eyes	Just one eye
Feline hyperesthesia syndrome	Daily	Weekly, monthly or less often than monthly
Feline upper respiratory infection	At the moment	Less than half a year ago or more than half a year ago
FeLV	Symptomatic	Asymptomatic
FIP	Any	-
FIV	Severe or minor symptoms	Asymptomatic
Food allergy	-	One or multiple symptoms selected: skin symptoms, ear redness/itching/infection, gastrointestinal symptoms
Gingivitis	At the moment	Less than half a year ago or more than half a year ago
HCM	Symptomatic	Asymptomatic
Heart murmur	-	Any

Hip dysplasia or other deformation of bones/joints	Minor or major symptoms	No symptoms
Hyperthyroidism	Causing symptoms at the moment	Caused symptoms less than half a year ago or more than half a year ago
Idiopathic cystitis	Causing symptoms at the moment	Causing symptoms less than half a year ago or more than half a year ago
Internal/intestinal parasites	Less than a week ago	Less than half a year ago or more than half a year ago
Kidney failure	Causing symptoms at the moment	Causing symptoms less than half a year ago or more than half a year ago
Mammary tumour	Symptomatic	Asymptomatic
Missing tear duct	Any	-
Nystagmus	At the moment and affecting life of the cat	Less than half a year ago or more than half a year ago and not affecting life of the cat
Osteoarthritis or other joint pain	One or multiple symptoms selected: withdraw/hide, playing with other cats decreased, limping, reluctant to jump up/down, reluctant to move, decreased grooming, more irritable	Selected no symptoms
Protozoans	Less than a week ago	Less than half a year ago or more than half a year ago
Repetitive constipation	Constantly	Weekly, monthly or less than monthly
Repetitive diarrhea	Daily	Several times a week, once a week or less than once a week
Repetitive vomiting	Daily	Weekly or less than once a week
Respiratory infection	At the moment	Less than half a year ago or more than half a year ago
Seborrhoea	At the moment	Less than half a year ago or more than half a year ago
Stomatitis	At the moment	Less than half a year ago or more than half a year ago
Tail kink	Affecting the behaviour (free text field)	Not affecting behaviour (free text field)
Tooth resorption	Causing symptoms or noticed at the moment	Causing symptoms or noticed less than half a year ago or more than half a year ago
Undescended testis	-	Any
Urinary tract infection	At the moment	Less than half a year ago or more than half a year ago
Uterine infection	Less than a month ago	A month - 6 months ago or more than 6 months ago
Unlisted diseases and health problems	Free text field. Severe disease or chronic health issue causing symptoms at the moment. Classified case-by-case similarly than listed diseases.	Free text field. Disease or other health issue not causing symptoms at the moment. Classified case-by-case similarly than listed diseases.

Table S5. Results of 5-fold cross validation process for the final models of fearfulness, aggression toward humans, and excessive grooming, related to Table 1.

	Fearfulness		Aggression toward humans		Excessive grooming	
Variable	% present in best models	relative influence	% present in best models	relative influence	% present in best models	relative influence
Age	base	3.37	base	0.32	base	8.22
Sex	base	0.55	base	0.24	base	0.16
Breed	base	19.94	base	15.69	base	8.60
Fearfulness			100	18.12	100	8.33
Aggression toward humans	100	13.56			100	19.97
Activity/playfulness	80	3.80	100	5.55	40	4.29
Sociability toward cats	0	6.37	100	30.36	40	4.92
Sociability toward humans	100	18.44	100	6.69	100	11.05
Litterbox issues	100	6.27	100	3.21	100	16.61
Excessive grooming	100	11.69	100	13.12	0	
Other cats in household	100	1.43	100	1.11	100*	0.76
Acquisition place	100	3.00	100	1.04	0	0.00
Main reason for getting the cat	100	2.38	40	0.14	20	0.00
Type of outdoor access	100	3.24	0	0.31	100	2.53
Socialization to humans	100	1.41	0	0.18	0	0.08
Hobby	100*	0.50	60	0.02	0	0.00
Hormonal status	100*	0.70	0	0.00	0	0.00
Large scratching trees	60	0.59	80	0.75	20	0.08
Previous cat experience/ownership	60	1.35	40	0.04	20	0.70
Feeding style	40	0.08	20	0.07	0	0.00
Food types	20	0.57	20	0.15	0	0.16
Time since last vet visit	0	0.03	100	1.44	60	0.36
Small scratching trees	0	0.07	60	0.17	20	0.12
Siblings	0	0.09	40	0.59	20	0.32
Socialization to animals	0	0.01	20	0.11	0	0.02
Health problems	0	0.13	0	0.16	100	11.03
Playtime frequency	0	0.36	0	0.34	40	1.53
Days the cat is left alone during the week	0	0.06	0	0.08	0	0.16
Sex * Hormonal status	0		0		0	
Acquisition place * Socialization to animals	0		0		0	
Acquisition place * Socialization to humans	0		0		0	
Socialization to humans * Socialization to animals	0		0		0	
Previous cat experience/ownership * Other cats in household	0		0		0	
Playtime frequency * Age	0		0		40	

Time since last vet visit *			
Health problems	0	0	60
Health problems * Age	0	0	80

* = variable was not included in the final model because a low relative influence

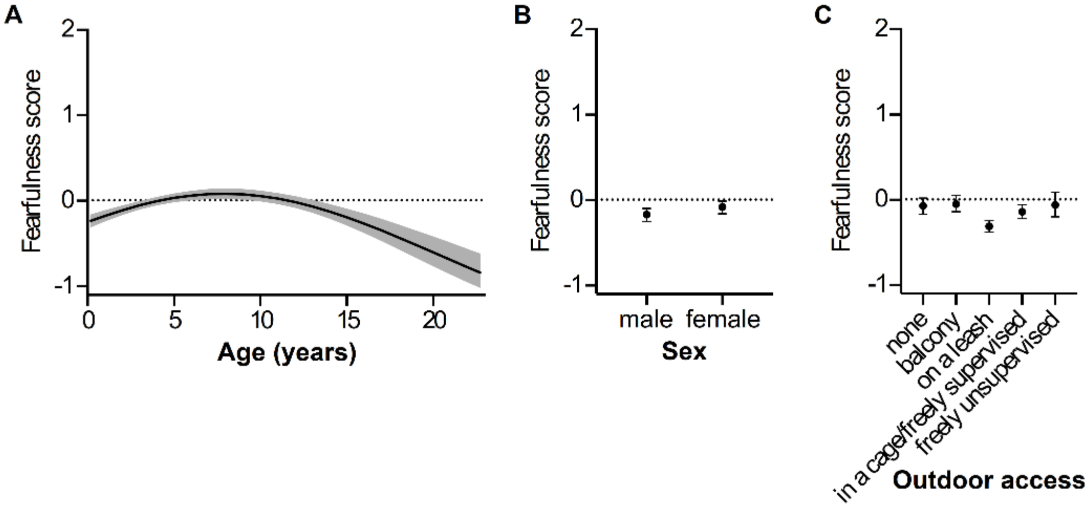

Figure S1. Demographical and environmental factors associated with fearfulness, related to Figure 1. Associations of the age (A), sex (B), and type of outdoor access (C) with fearfulness in the generalized linear model. Grey area and error bars indicate 95% confidence limits. *N* = 3255.

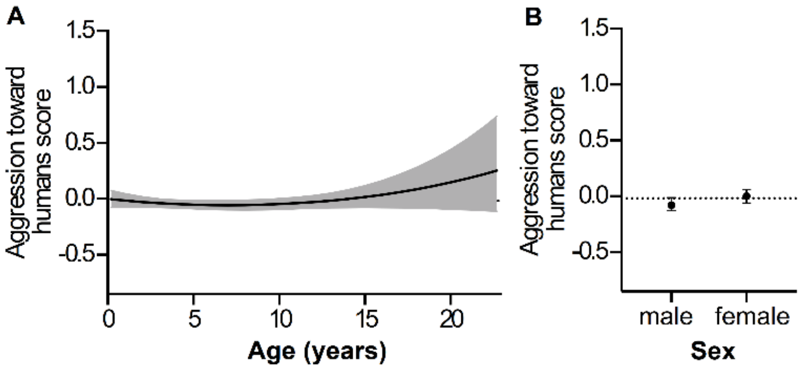

Figure S2. Demographical factors associated with aggression toward humans, related to Figure 3. Associations of the age (A) and sex (B) with aggression toward humans in the generalized linear model. Grey area and error bars indicate 95% confidence limits. *N* = 3255.

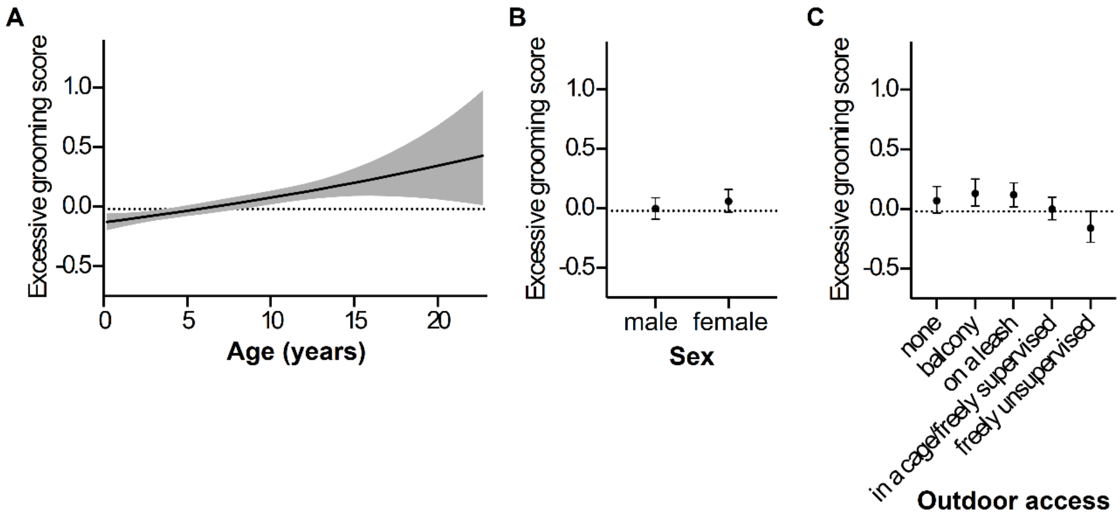

Figure S3. Demographical and environmental factors associated with excessive grooming, related to Figure 5. Associations of the age (A), sex (B), and type of outdoor access (C) with excessive grooming in the generalized linear model. Grey area and error bars indicate 95% confidence limits. *N* = 3255.
